# A Scalable Polio-EPI Synergy Model for Urban Immunization: Coverage Gains Following Workforce Integration in Lahore, Pakistan

**DOI:** 10.3390/vaccines14020167

**Published:** 2026-02-11

**Authors:** Imtiaz Hussain, Naeem Majeed, Ali Jan Khan, Ahmad Khan, Muhammad Umer, Uzair Ansari, Zara Ansari, Hamza Fawad, Sajid Bashir Soofi

**Affiliations:** 1 Centre of Excellence in Women and Child Health, Aga Khan University, Karachi 74800, Pakistan; imtiaz.hussain@aku.edu (I.H.); ahmad.sherali@aku.edu (A.K.); muhammad.umer@aku.edu (M.U.); uzair.ansari@aku.edu (U.A.); 2 Department of Pediatrics & Child Health, Aga Khan University, Karachi 74800, Pakistan; 3 SPHERE Consulting, Lahore 54000, Pakistan; naeem@sphereconsulting.services; 4 Primary & Secondary Healthcare Department, Government of Punjab, Lahore 54700, Pakistan; alijankhan@yahoo.com; 5 Independent Consultant Public Health Research, Lahore 54000, Pakistan; zara.ansari1@gmail.com; 6 Acasus, Lahore 5400, Pakistan; hamza.fawad@acasus.com

**Keywords:** routine immunization, urban health system, health workforce integration, polio eradication initiative, Pakistan

## Abstract

**Background:** Large urban centers in low- and middle-income countries (LMICs) often have persistent pockets of under-immunized children, despite higher overall vaccination coverage than rural areas. Lahore, a megacity in Pakistan, had the lowest rate of fully immunized children in Punjab province as of 2022 (70%), partly due to challenges in its urban slums. In 2023, an innovative intervention was implemented, utilizing Pakistan’s extensive polio eradication workforce to identify and reach children who were missing routine vaccinations. **Objective:** The objective was to assess changes in routine immunization coverage during a pre–post evaluation period in which polio campaign workers were engaged to support routine immunization among under-immunized urban populations in Lahore. **Methods:** A special outreach strategy engaged polio vaccination teams to conduct door-to-door visits of children aged 12–23 months, recording each child’s routine immunization status. These data were integrated into the electronic health system and provided to Expanded Programme on Immunization (EPI) staff for targeted follow-up. Two cross-sectional household surveys of caregivers of children aged 12–23 months were conducted: a 2022 baseline survey and a 2023 follow-up survey conducted six months after implementation. Both surveys used two-stage cluster sampling and WHO-standard immunization coverage methods, with vaccination status verified using cards or caregiver recall. **Results:** A total of 773 children were surveyed at baseline and 780 at endline. Full immunization coverage increased from 69.8% (CI: 64.13–74.98) to 85.1% (CI: 81.01–88.51). Partial immunization declined from 26.9% (CI: 22.37–31.92) to 14.5% (CI: 11.27–18.50), and the proportion of children not vaccinated at all dropped from 3.3% (CI: 1.92–5.60) to 0.3% (CI: 0.11–0.98). Penta-3 coverage improved from 83.2% (CI: 78.65–87.04) to 94.1% (CI: 91.15–96.07), and Measles 1 from 76.9% (CI: 71.80–81.40) % to 92.1% (CI: 88.71–94.56). Immunization card retention increased from 69.9% (CI: 64.15–75.16) to 84% (CI:81.19–86.94). Improvements were observed across all socio-demographic groups, with a higher impact in peri-urban clusters and low socio-economic groups, and all remained statistically significant. **Conclusions:** Our findings showed improvements in routine immunization coverage in urban Lahore between 2022 and 2023. This period coincided with district-wide implementation of a polio worker outreach strategy as well as the broader post-COVID-19 recovery of immunization services. This study lacked a control group; therefore, the findings indicate a temporal association occurring during the post COVID-19 recovery period, rather than definitive evidence of causal impact. Nonetheless, integrating the workforce of the polio program into routine immunization could be a promising programmatic strategy to close immunization gaps in urban areas.

## 1. Introduction

Routine childhood vaccination is one of the most cost-effective public health interventions, preventing an estimated 2–3 million child deaths globally each year [1]. However, achieving and maintaining high vaccination coverage in densely populated urban areas has become increasingly challenging as the world urbanizes. As of 2019, 55% of the world’s population lives in urban areas, a figure expected to reach 68% by 2050 [2]. A significant portion of urban growth is occurring in low- and middle-income countries, often concentrated in informal settlements (slums) characterized by poverty, overcrowding, and limited access to healthcare [3]. While average immunization rates in cities tend to exceed those in rural areas, this so-called “urban advantage” is blunted by large intra-urban disparities. Many of the remaining zero-dose children (those who have never received any routine vaccine) reside in urban slums and peri-urban communities [3]. These pockets of under-immunized children are at heightened risk for vaccine-preventable disease outbreaks and contribute to persistent inequities in health outcomes.

Multiple factors underlie immunization gaps in urban settings. Demand-side barriers include lack of awareness or misinformation about vaccines, fear of side effects, time constraints for working mothers, and distrust of government services [4,5,6]. In socially marginalized urban communities, cultural and religious misconceptions or reliance on traditional healers can also lead to vaccine hesitancy [4,6]. Supply-side barriers in cities often relate to health system shortcomings: inadequate numbers of outreach clinics in informal settlements, poor attitude or training of health staff, fragmented record-keeping for highly mobile populations, and strains on workforce capacity due to frequent supplemental immunization activities [4,7]. The separation of vertical health programs can further compound these issues. For example, polio eradication campaigns repeatedly canvass urban areas, sometimes causing “campaign fatigue” in communities. Yet, the data and resources from these campaigns have not always been utilized to strengthen routine immunization services [7].

Pakistan offers a compelling context to explore the integration of immunization efforts in urban areas. The country’s Expanded Programme on Immunization (EPI) has gradually improved national coverage (estimated 66% fully immunized children in 2018 and 76.5% by 2022), but progress has been uneven [4,8,9,10]. In the urban areas of Sindh, for example, only 63% of children were fully immunized in 2018, with coverage in large peri-urban slums even lower, highlighting persistent implementation challenges [4]. Punjab has historically performed better overall, yet its capital city, Lahore, has consistently lagged behind several smaller districts in recent surveys [11]. Lahore is Pakistan’s second-largest city by population (approximately 11 million) and has a mix of affluent areas and numerous informal settlements. According to a 2018 provincial survey, only about 67% of Lahore’s children aged 12–23 months were fully immunized, indicating substantial coverage gaps in this major metropolis [12]. By 2022, a third-party coverage evaluation survey found that Lahore had the lowest full immunization coverage (FIC) among the 36 districts of Punjab, at approximately 70%, compared to the provincial average of 88.9% [11].

At the same time, Pakistan’s Polio Eradication Initiative (PEI) operates one of the largest immunization workforces in the world. Nationwide polio campaigns (National Immunization Days (NIDs)) are conducted multiple times each year, mobilizing over 400,000 frontline workers to go door-to-door vaccinating children under five with oral polio vaccine [13]. During these campaigns, polio teams enumerate all children under five years old in each household and are uniquely skilled at finding newborns and migrants. For years, the PEI and routine immunization programs worked in parallel with minimal coordination. However, there is growing recognition that the massive PEI infrastructure could be leveraged to strengthen routine immunization delivery, especially in hard-to-reach urban communities [10]. Integrating efforts can be mutually beneficial; improvements in routine immunization raise population immunity against polio, and polio campaigns can achieve higher acceptance if coupled with broader health services [10]. Indeed, recent strategy frameworks in Pakistan explicitly call for optimizing the “synergy” between PEI and EPI to achieve universal coverage [14].

In late 2022, the Government of Punjab launched a special initiative in Lahore to rapidly improve routine immunization outcomes by utilizing the polio campaign workforce. This initiative was prompted by Lahore’s poor immunization performance and aimed to test a novel approach to close the urban immunization gap. Polio campaign teams, already accustomed to visiting every house, were given an additional task during their rounds: to list all children under 2 years of age and record each child’s vaccination status (whether up to date, missing doses, or never vaccinated). These lists, including detailed addresses and parent contact information, were then provided to Lahore’s routine immunization staff (vaccinators and supervisors) in the EPI. The data were digitized and entered into the Health Management Information System (HMIS)—the Punjab’s Electronic Medical Records (EMR), effectively creating an updated nominal roll of children who are due or defaulted for every union council in the city. Equipped with this information, vaccinators could conduct targeted follow-up visits to families of under-immunized children in their catchment areas. The performance of vaccinators in reaching these children was monitored through a geographic information system (GIS) that tracked their outreach, and progress was reviewed in daily and weekly meetings by district health managers. The initiative represented an unprecedented collaboration between the polio eradication infrastructure and the routine immunization program in an urban district.

This study assesses the changes in routine immunization coverage in Lahore during pre-post evaluation conducted after the implementation of the intervention. We compare key immunization coverage indicators before (Third-Party Verification of Immunization Coverage Survey-TPVICS 2022) and after (Lahore Coverage Survey 2023) the implementation of the polio workforce-leveraged strategy. We hypothesized that the intervention would substantially increase the proportion of fully immunized children and reduce the number of zero-dose (unvaccinated) children in Lahore. The study also examines improvements in specific vaccine coverage (e.g., third-dose pentavalent and measles) and whether gains were equitable across urban subpopulations.

## 2. Materials and Methods

### 2.1. Study Design and Setting

We conducted a pre- and post-evaluation study, using cross-sectional household surveys at two time points: before the intervention (baseline) and following the intervention (endline). The study was conducted in the district Lahore in Punjab, Pakistan, where public immunization services operate through fixed facilities and outreach activities led by government vaccinators under the supervision of the District Health Office.

The intervention, which involved engaging polio workers to support routine immunization services, began in February 2023 and continued throughout the rest of the year. Data from the second round TPVICS, conducted in April–June 2022, served as the baseline, while data from a comparable survey conducted in August–September 2023 were used for the endline assessment. Both surveys targeted children aged 12–23 months, the standard age range for evaluating infant immunization coverage. It is important to note that the intervention extended beyond the endline survey timeline; therefore, the findings reflect progress achieved during the initial six months of implementation.

### 2.2. Sampling Procedure

A two-stage cluster sampling design was employed for both baseline and endline surveys, following World Health Organization (WHO) EPI coverage survey guidelines [15]. In stage one, 64 primary sampling units (PSUs) were selected randomly from the district. The PSUs corresponded to geographic clusters (urban blocks or villages) defined by the Pakistan Bureau of Statistics.

In stage two, households within each selected cluster were enumerated to identify those with eligible children (aged 12–23 months). Using the fresh listing, 13 households with at least one eligible child were randomly selected from each cluster for interview in the baseline survey, and the same procedure was followed in the endline survey with an updated household listing for each cluster. If a household had more than one eligible child, one was randomly chosen as the index child for immunization assessment to maintain equal weighting.

### 2.3. Sample Size Estimation

The sample size was determined according to the WHO 2018 coverage manual guidelines to ensure representativeness at the district level [15]. The survey was designed to achieve a precision of ±5.5% for district-level coverage estimates, assuming an expected coverage of 50–70% and a design effect of 2.5% and a 95% confidence level. Based on these parameters, a minimum of ten respondents aged 12–23 months were required per cluster. To account for potential non-response, 13 households per cluster were targeted, yielding a total sample size of 832 children aged 12–23 months across the district.

### 2.4. Data Collection Procedure

The surveys were conducted at the household level with the child’s primary caregiver (usually a parent). Trained survey teams visited each selected household and obtained informed consent verbally. A structured questionnaire, adapted from the standard WHO immunization coverage survey tools, was used to collect data on the child’s sex, date of birth, caregiver’s education, household socio-economic indicators (e.g., assets for wealth quintile classification), and vaccination history. Caregivers were asked to provide the child’s immunization card if available; interviewers recorded dates of each vaccine from the card. If no card was produced, the caregiver’s verbal recall of each vaccine dose received was recorded. The questionnaire also included questions about whether any health worker involved in vaccination had visited the family in recent months, to assess exposure to the intervention. Field supervisors reviewed completed forms for accuracy and consistency.

### 2.5. Intervention Description

While the surveys themselves did not implement an intervention, the endline survey was explicitly conducted after a six-month intervention in Lahore’s routine immunization program. The intervention involved: (1) Polio worker engagement: During polio vaccination campaigns, workers compiled lists of children under two years and their immunization status (categorized as “vaccinated up-to-date,” “due” for eligible doses, or “defaulter” missing many doses). (2) Data integration: These lists were handed to union-council supervisors, then digitized into the Punjab’s electronic medical record (EMR) database (also known as HMIS for primary care). Each government vaccinator serving a specific area could access a list of children’s names and addresses who were due/defaulter for vaccines in that area. (3) Targeted outreach: Vaccinators were instructed and supported to follow up with these families during routine work and outreach sessions, to either vaccinate the child or counsel parents to visit the clinic. Additional vaccine supply was ensured so that vaccinators could provide any missing doses on the spot. (4) Monitoring: The movement of vaccinators during outreach was tracked via the EMR application, ensuring they visited the mapped households. Progress reports (number of defaulters vaccinated) were reviewed in frequent meetings at the district and provincial levels on a daily, weekly, and fortnightly basis, with various levels of participation. This intensive approach was sustained for several immunization cycles. It is important to note that no changes were made to the immunization schedule or vaccine supply; the difference lay in more efficient identification and follow-up of unvaccinated children using the polio workforce’s reach.

### 2.6. Outcomes and Definitions

The primary outcome was the proportion of children 12–23 months who were fully immunized according to Pakistan’s EPI schedule. In Pakistan, a child is considered fully immunized by 12 months of age if they have received: one dose of Bacille Calmette–Guérin (BCG) vaccine, four doses of oral polio vaccine (OPV), and three doses of pentavalent vaccine (diphtheria-pertussis-tetanus-hepatitis B-Haemophilus influenzae type b), three doses of pneumococcal conjugate vaccine (PCV), and one dose of inactivated polio vaccine (IPV) and one dose of measles-containing vaccine (measles-rubella) by 9 months. Two doses of the Rota virus vaccine, the second measles vaccine dose, and a typhoid conjugate vaccine are given in the program, but were not counted in the definition of “fully immunized” for this age group. A child missing any of the required doses by 12 months was categorized as partially immunized. A child who had received no routine vaccines at all (zero doses) was classified as unimmunized. Secondary outcomes included coverage rates for individual antigens (e.g., percentage receiving BCG, OPV3, Pentavalent-3, first measles, etc.), immunization card retention rate (as a proxy for caregiver record-keeping and health system contact), and equity of coverage by gender, urban/rural residence (Lahore district includes some peripheral rural areas), maternal education level, and household wealth quintile.

### 2.7. Data Analysis

Survey data were analyzed using STATA (Version 18.0), accounting for the complex survey design (clusters and sampling weights). Sampling weights were applied to adjust for the probability of selection and non-response for each child, ensuring that the results are representative of the entire district population of children aged 12–23 months. Weighted coverage proportions and 95% confidence intervals were computed for all key indicators at baseline and endline. We compared the 2022 and 2023 coverage estimates to assess changes; z-tests for two-sample proportions (adjusted for clustering) were performed to determine if changes were statistically significant. Additionally, stratified analyses were performed to evaluate coverage differences across subgroups (gender, urban vs. peri-urban, lowest vs. highest wealth quintile). In this paper, we present descriptive comparisons rather than a formal regression, given the pre/post nature of the data. The threshold for significance was *p* < 0.05 for changes in coverage.

## 3. Results

### 3.1. Survey Population and Household Characteristics

Of the 832 households sampled in the 2022 baseline survey, 784 were successfully interviewed, yielding a response rate of 94%. In the endline survey (2023), all 832 sampled households were approached, and interviews were conducted with 737 households, yielding a response rate of 88.6%. 773 children aged 12–23 months were covered at baseline, and 780 at endline. The mean age of enrolled children was approximately 18 months at the time of data collection. Of the children surveyed at baseline, 52% were female, and 48% were male; at endline, 48% were female, and 52% were male (Table 1). Urban residents constituted 84.1% in the baseline and 79% in the endline survey of the sample, while 15.9% and 21% respectively (baseline and endline) lived in the periphery of the district, categorized as peri-urban areas. The children were nearly evenly distributed across socioeconomic quintiles (by design, each wealth quintile comprised ~20% of the sample), indicating no significant bias toward richer or poorer households in both survey rounds. A change in maternal education levels was observed between the two survey rounds. Mothers who had no formal schooling declined (24.6% to 16.9%), while attainment at primary (3.7% to 12.1%), middle school education (6.86% to 10.77%), and secondary (18.4% to 25.1%) levels improved. On the other hand, the maternal education level at higher secondary education or above decreased (46.4% to 35.1%).

### 3.2. Immunization Coverage Levels

Routine immunization coverage in Lahore improved markedly following the intervention. In 2022, the proportion of children aged 12–23 months who were fully immunized was 69.8% (CI: 64.13–74.98). By 2023, this proportion had increased to 85.1% (CI: 81.01–88.51), representing a statistically significant gain of 15.3 percentage points (*p* < 0.001). This indicates that the majority of children in Lahore have received all basic vaccines in their first year of life. Correspondingly, the percentage of partially immunized children declined from 26.8% (CI: 22.37–31.92) to 14.5% (CI: 11.27–18.50) in the endline. Similarly, the proportion of unimmunized children reduced to 0.3% (CI: 0.11–0.98) from 3.3% (CI: 1.92–5.60) at baseline. In absolute terms, the number of zero-dose children found in our endline sample was just 3 out of 780, compared to 26 out of 807 at baseline (Table 2).

Coverage of the third dose of pentavalent vaccine (Penta-3)—a critical measure of the immunization program’s reach increased to 94.1% (CI: 91.15–96.07) in the endline from 83.3% (CI: 78.65–87.04) in the baseline. This indicates that 9 in 10 infants are now receiving the complete pentavalent vaccine. First-dose measles coverage also improved to 92.1% (CI: 88.71–94.56) from 76.9% (CI: 71.80–81.40) at baseline, reflecting timely vaccination by the end of the first year. Even for vaccines introduced into the schedule in recent years, coverage was substantial. For example, the proportion of children receiving the second dose of IPV (introduced in 2021) reached 76.4% (CI: 71.86–80.44), and the typhoid conjugate vaccine (TCV, introduced in late 2021) reached 83.9% (CI: 79.57–87.42). However, the coverage of hepatitis B declined to 65.5% (CI: 59.36–71.21) coverage at the endline compared to baseline (69.3%; CI: 63.54–74.54), and remains the lowest among antigens, suggesting that one-third of infants in Lahore still miss the timely birth dose (Table 2).

Immunization card retention among caregivers improved, both as an outcome and an enabler of better coverage. At the endline, 84.3% (CI: 81.19–86.94) of children had their vaccination card available and seen by the surveyor, compared to 70% (CI: 64.15–75.16) at baseline (Table 2). High card availability suggests that caregivers are more engaged in maintaining vaccination records, which facilitates accurate dose verification. It also likely reflects the impact of health worker engagement, as vaccinators reached out to defaulting families, they may have distributed or updated immunization cards, encouraging families to hold onto these records.

### 3.3. Coverage by Subgroup

Our survey findings showed improvements in coverage across nearly all geographic areas in the district by the endline. Though some disparities remained, the gains were broad-based. Since the intervention was implemented district-wide, both urban and peri-urban localities in Lahore experienced measurable benefits. In the district’s urban areas, FIC reached 86.3% (CI: 82.44–89.42) at the endline, compared to 70.3% (CI: 64.47–75.52). In the per-urban areas, it increased from 67.6% (CI: 51.45–80.37) to 81.2% (CI: 67.19–90.08) (Table 3). Similarly, the coverage for Penta 3 and Measles 1 improved in urban and peri-urban areas from baseline to endline (Table S1).

Our findings showed that FIC increased for both male (68.5%; CI: 61.55–74.74 to 83.1%; CI: 78.03–87.20) and female (71.2%; CI: 64.67–77.00 to 87.3%; CI: 82.22–91.15) children between the two rounds (Table 3). By the endline, coverage for nearly all antigens (e.g., Penta-3, Measles 1) increased and remained higher among girls. For example, Pentavalent-3 coverage reached 94.6% (CI: 90.54–96.93) compared to 93.6% (CI: 90.51–95.74) among boys (Table S1). Similarly, FIC improved across all maternal education groups; however, the gains were more pronounced among mothers with no formal schooling (45.7%; CI: 36.24–55.48 to 72.1%; CI: 60.23–81.51) and those with primary (59.1%; CI: 40.48–75.39 to 84.4%: CI: 74.05–91.08) and middle level schooling (61.5%: CI: 49.89–71.88 to 91.7%: CI: 83.03–96.10) (Table 3). Likewise, improvements in coverage for Penta 3 and Measles 1 were more pronounced among mothers without formal education and those with primary- and middle-level schooling (Table S1).

Immunization coverage gains were also evident across all wealth quintiles; however, children from the poorest households still have slightly lower coverage than those in the richest quintile. At endline, FIC in the poorest quintile reached 76.5%; CI: 65.81–84.69, compared to 87.7%; CI: 81.14–92.22 in the richest quintile. At baseline, these figures were approximately 49%; CI: 38.57–59.37 and 81.5%: CI: 72.08–88.32, respectively, indicating a substantial improvement in the poorest quintile, by over 27 percentage points, and narrowing the equity gap. FIC in the middle and rich quintiles improved from 73.1%: CI: 64.88–79.87 to 87.5%; CI: 81.50–91.87 and from 73.5%: CI: 63.61–81.58 to 90.2%; CI: 84.16–94.16, respectively, reflecting broad progress across socioeconomic groups, notably, even in the poorest communities (Table 3). Coverage for specific vaccines across the wealth quintiles reflected similar patterns: For example, Penta 3 coverage in the poorest quintile increased from 66.11%: CI: 55.96–74.96 to 85.8%: CI: 74.14–92.80, and in the rich quintile it increased from 83.1%: CI: 74.70–89.13 to 96.8%: CI: 92.93–98.64. Likewise, Measles 1 coverage improved from 56.3%: CI to 82.7%: CI in the poorest group, and it increased from 77.5%: CI to 94.3%: CI in the rich group (Table S1). However, the existing coverage gap is larger in the lower quintiles than in the higher quintiles. This gap likely reflects differences in healthcare-seeking for follow-up visits, indicating the need for targeted strategies to improve follow-up in poorer localities to boost second-year vaccination uptake in these pockets.

Coverage across all subgroups improved from baseline to endline; however, differences between subgroups were not statistically significant. The subgroup analyses were exploratory and were not adjusted for multiple comparisons or potential confounders. These findings need to be interpreted cautiously, given the exploratory nature of the analysis and limited statistical power.

## 4. Discussion

This study demonstrates that creating synergy between the polio eradication workforce and the routine immunization system through systematic data sharing, targeted follow-up, and sustained monitoring can deliver rapid and meaningful gains in immunization coverage and reduce the equity gap in a challenging urban environment. Improvements in FIC & partial vaccination & unimmunized children reflect that the intervention was successful in reaching previously missed and underserved children and in substantially strengthening routine immunization performance in the district. In other words, thousands of children in Lahore who would have been completely unprotected are now at least partially vaccinated. Furthermore, improvement in the third-dose pentavalent coverage to 94% reflects sustained dose completion rather than isolated uptake, indicating a strengthened delivery system rather than temporary campaign effects.

The trajectory of improvement contrasts sharply with coverage trends in Lahore and across the region over recent years. In the aftermath of the COVID-19 pandemic, FIC in Lahore remained stagnant or even declined, mirroring patterns observed across South Asia [16]. Between 2020 and 2024–2025, FIC trends in Pakistan showed an uneven pattern of post-pandemic recovery. Nationally, the coverage declined moderately from 76.4% to 73% [17,18]. At the provincial level, Punjab experienced a substantial decline (89.9% to 79%), while Sindh showed a modest improvement (61.1% to 66%) and Khyber Pakhtunkhwa demonstrated marginal progress (68.4% to 69%). Balochistan, on the other hand, reflected a higher progress from 37.4% to 54% during the same period [17,18].

Nonetheless, a modest recovery in routine immunization was observed across South Asian countries since 2022. WHO/UNICEF estimates indicate that DTP3 coverage increased between 2020 and 2024 in several South Asian countries, including India (85% to 94%), Nepal (84% to 97%), Bhutan (95% to 98%), the Maldives (98% to 99%), and Pakistan (80% to 87%) [19]. Against this backdrop of regional recovery, the rebound to 85% FIC in Lahore, exceeding pre-pandemic levels, suggests that the improvements observed in the study setting occurred (yet exceeded) within a broader post-COVID recovery in routine immunization services rather than in isolation. Our study provides quantitative evidence for what was previously anecdotal: integrating PEI and EPI can yield “quick wins” for immunization coverage. As demonstrated in Khyber Pakhtunkhwa, integrating the PEI field workforce and data systems into the EPI platform has resulted in marked gains in routine antigen coverage [10]. The Lahore experience adds to this evidence in a large, diverse urban district and would thus support its applicability beyond areas traditionally perceived as high-risk or rural.

The findings from this study align with the Immunization Agenda 2030, ensuring that every district achieves higher coverage of essential vaccines and reduces zero-dose inequities [20]. Our findings strengthen the case for using targeted, data-driven outreach strategies to close persistent gaps in densely populated cities across the region. The findings from this study are consistent with evidence from Nigeria, Afghanistan, and priority Global Polio Eradication Initiative (GPEI) countries, where redeployment of polio staff has been associated with improvements in defaulter tracing, microplanning, and improving the coverage of the third dose of diphtheria, tetanus, and pertussis (DTP3) and other antigens [21,22,23]. Pakistan-specific analyses have likewise argued that operational alignment between the EPI and PEI is essential to strengthen routine vaccination and achieve eradication goals [24]. Our study goes a step further by providing district-level empirical evidence that systematic integration of polio field staff directly into routine activities improves follow-up of defaulter children, card retention, and immunization coverage. In doing so, it demonstrates that synergy between vertical and routine systems can move beyond policy aspiration to operational reality.

A key mechanism of impact in our intervention seems to be the improved identification and tracking of defaulter children. In urban settings, EPI vaccinators often struggle to know how many children are in their catchment areas, who they are, and which doses they are missing, especially with high population mobility and the influx of migrants, by deploying polio campaign workers to register every child under two years of age, including name, address, and vaccination status, the program created, for the first time, a complete nominal registry of the target population. This approach is consistent with findings from other studies in similar contexts; for instance, a study in peri-urban Karachi noted that accurate population denominators and tracking systems are crucial for improving coverage in slum settings [4]. Similar experiences have been documented elsewhere; GPEI-supported staff in other countries have been shown to conduct micro-census work, defaulter tracing, and household mapping in highly mobile or poorly enumerated communities, thereby strengthening routine immunization (RI) delivery [21,22]. Nigeria’s National Stop Transmission of Polio (NSTOP) program also demonstrated that polio officers used mapping tools and household registries to improve routine vaccination and follow-up in dense urban areas [23]. Together, this evidence supports the point that leveraging polio personnel to enhance population verification and defaulter follow-up can be a major driver of the improvements observed in our study. Operationally, integrating the “house-to-house” strategy from polio campaigns into the routine immunization system enabled the district health authorities in Lahore to align RI and outreach sessions far more precisely than was previously possible.

Enhanced monitoring and accountability were integral to our intervention. The intervention didn’t stop at handing lists to vaccinators; it also tracked whether those children were subsequently vaccinated using GIS. Regular review meetings created a culture of accountability and even healthy competition: field staff were aware that their performance (how many due children they converted to fully vaccinated) was being closely watched by supervisors. This likely motivated vaccinators to follow through on outreach activities that might previously have been neglected. Such performance-management interventions, essentially strengthening the health workforce through supervision and feedback, are known to improve service delivery [7,25,26,27,28]. These strategies mirror systematic accountability frameworks used in polio programmes, which have been associated with improved programme performance and reduced missed children [27].

Beyond system performance, the intervention appears to have influenced caregiver behaviour. Home visits reframed routine immunization as a priority rather than a passive service. When polio workers visited specifically to check routine vaccination status, they delivered a message to families that routine immunization is important. Some caregivers told vaccinators they didn’t realize specific doses were due; the visit served as a reminder and prompted them to act. The increase in immunization card retention from 70% to 84% suggests that caregivers became more conscious of maintaining vaccination records, likely influenced by these engagements. Evidence from other settings consistently shows that regular home visits, reminder systems, and interpersonal communication significantly increase immunization uptake and record retention [7,29]. Lahore’s approach, by institutionalizing a reminder system through polio visits, appears to have addressed at least part of this demand-side gap.

Importantly, the intervention contributed to narrowing vaccination coverage inequities. Coverage gains were more pronounced among children from the poorest households and those born to mothers with no formal education, indicating that the initiative benefited groups historically most likely to be missed. Nonetheless, socioeconomic and educational gradients still influence the immunization outcomes; children from poorer households and those with less educated mothers still have somewhat lower FIC. Additional strategies, such as engaging community influencers, offering more flexible clinic hours, and providing tailored counselling, may help convert partial immunization to full immunization among hesitant or hard-to-reach families [4,30]. These findings imply that the intervention not only increased total coverage but also promoted equity by targeting children least likely to access routine services through conventional delivery models.

The intervention has potential implications for polio eradication itself. Lahore has historically been at risk for poliovirus circulation due to migrant influx, but higher IPV and OPV coverage could improve population immunity. Our findings indicate that IPV2 coverage was 76.4% in Lahore, representing significant progress since its introduction. High uptake of IPV, along with OPV through campaigns, helps close immunity gaps. The GPEI has long emphasized strengthening routine immunization in polio reservoirs as a key action to “finish the job” [10]. In this sense, the Lahore initiative represents a win-win: polio program resources helped improve routine vaccines and routine coverage, in turn strengthening the polio endgame.

When compared with other urban immunization interventions, the magnitude and pace of improvement observed in Lahore are at the higher end of what has been documented. For instance, a study in Dhaka’s slums reported an increase in FIC from 43% to 70–85% after an intervention package that included evening clinics and community volunteers, a finding comparable in scale to ours. Lahore’s approach differed in that it largely relied on an existing workforce rather than creating new structures, suggesting potential cost-efficiency [30]. A systematic review by Nelson et al. (2016) found that interventions such as home visits, community mobilization, and extended service hours were effective in urban contexts, but very few documented cases used polio campaign modalities [7]. Our study fills that gap by providing evidence for the effectiveness of such an approach. It underscores that bridging vertical programs (polio) with horizontal services (routine immunization) can address both supply and demand issues simultaneously: supply is improved by active outreach and data use, and demand is nudged by repeated community contact and trust-building through familiar workers.

Regarding generalizability, what was implemented in Lahore could be adapted to other major cities in Pakistan and beyond. This mainly depends on the strong political will, effective coordination between PEI, EPI, and local government, and efficient data systems that can integrate household-level information into routine planning. Cities with ongoing polio campaigns could emulate this approach to identify zero-dose children through routine programs. Even in cities with no active polio campaigns, the concept of door-to-door visits could be implemented through community health workers/vaccinators or by organizing regular drives targeting routine immunization. However, such intensive activity requires sufficient funding, including workers’ incentives and transport support. In Lahore, some government operational funds were reallocated to support vaccinators in outreach (e.g., transport for vaccine deliveries and field visits). Ensuring that RI programmes are adequately financed to sustain outreach will be critical [4]. Encouragingly, the National Immunization Policy 2022 emphasizes strengthening coordination and operational synergy between the PEI and the EPI [31]. By committing to enhanced collaboration, shared micro-planning, and harmonized service delivery, particularly for zero-dose and underserved urban populations, the policy creates the conditions for successful models, such as those piloted in Lahore, to be sustained and scaled in other contexts in the country.

This study has several strengths, including high response rates in both survey rounds, comparable baseline and endline demographic profiles, and the use of card-verified vaccination data, which enhances accuracy. However, it is limited by its pre-post design and the absence of a control district, which restricts causal inference and prevents definitive attribution of the observed improvements solely to the intervention. As a result, the effect of other external factors cannot be fully excluded. Future studies should be accompanied by more rigorous evaluation designs to strengthen causal inference and assess long-term sustainability. Additionally, some reliance on caregiver reports in the absence of a vaccination card introduces potential recall bias, and the short observation window limits assessment of long-term sustainability. Our study also presents several subgroup analyses to assess changes in coverage across sociodemographic characteristics. Statistical adjustment for these multiple comparisons was not applied, and we acknowledge that conducting multiple subgroup comparisons may increase the risk of Type I error. Therefore, the associated *p*-values need to be interpreted with caution.

Given this, the sustainability of this approach needs to be considered. The findings need to be interpreted as reflecting early implementation effects observed within a six-month follow-up period, rather than definitive evidence of long-term gain. Global initiatives primarily fund the polio program in Pakistan. As polio eradication (hopefully) nears, there are plans to transition and integrate its assets into routine health services. Lahore’s experience provides a model for how that transition can tangibly improve outcomes, but it also relied on extraordinary monitoring efforts that might be hard to maintain long-term. The intensity of daily supervision and GIS tracking was feasible under emergency campaign mode; the health department will need to institutionalize these practices (perhaps at a lower frequency) to ensure coverage gains are sustained beyond the campaign context. In settings without a polio infrastructure, the core component of polio-EPI synergy, including household-level identification of missed children, data-driven microplanning, targeted outreach, and performance monitoring, can be adapted using existing community health workers or vaccinators.

Furthermore, we did not perform a formal cost-effectiveness analysis, which limits accurate quantification of incremental costs; however, the intervention depended mainly on reallocation of existing polio and routine immunization resources rather than new recurrent expenditures.

Despite these limitations, this study shows that integrating polio field staff into routine EPI activities can yield meaningful improvements in immunization coverage in an urban setting. Continued investment in such an integrated approach, alongside targeted support for marginalized populations, will be essential to sustain the gains, reach remaining underserved children, and further strengthen Pakistan’s routine immunization system.

## 5. Conclusions

Routine immunization coverage in urban Lahore increased substantially between the baseline (2022) and the endline (2023). This period coincided with both the implementation of the outreach strategy engaging the polio eradication workforce and the broader post-COVID-19 recovery of immunization services. As this pre–post evaluation did not include a control group, the observed improvements cannot be interpreted as evidence that the intervention caused the improvement in coverage, but rather as a temporal association that may reflect secular trends occurring during the recovery phase following the COVID-19 pandemic.

Despite these limitations, Lahore’s experience shows that engaging the resources of the polio program for routine immunization could be a feasible programmatic strategy to address coverage gaps in urban areas.

As polio assets transition, integrating them into routine immunization operations could enhance service delivery capacity, promote more equitable vaccination coverage, and strengthen health system resilience.

To sustain these gains and scale up the model, institutionalizing cooperation between EPI and the PEI including formalized data sharing, ongoing community engagement, and performance-based supervision will be essential.

## Figures and Tables

**Table 1:** Demographic characteristics of the population surveyed in the baseline and endline.

Variable	Baseline 2022	Endline 2023
Households sampled	832	832
Eligible Households successfully interviewed	784	737
Response rate (%)	94.23	88.58
Total eligible children (12–23 m) approached (N)	801	780
Total eligible children (12–23 m) interviewed (N)	773	780
**Sex distribution (%)**		
Female	52.15	48.33
Male	47.85	51.67
Mean age of children (months)	17.79	17.98
**Place of residence (%)**		
Peri-Urban	15.94	21.03
Urban	84.06	78.97
**Wealth quintile distribution (%)**		
Poorest	19.76	19.27
Poor	19.52	20.22
Middle	19.19	20.35
Rich	19.49	20.35
Richest	22.04	19.81
**Maternal education (%)**		
No formal schooling	24.62	16.92
Primary school	3.71	12.05
Middle school	6.86	10.77
Secondary (Matric)	18.43	25.13
Higher secondary or above	46.38	35.13

**Table 2:** Routine immunization coverage in district Lahore-baseline vs. endline.

Indicator	Baseline (2022)		Endline (2023)		Difference	*p*-Value
	N = 773	95% CI	N = 780	95% CI		
Fully immunized (all basic vaccines) **	69.83	64.13–74.98	85.15	81.01–88.51	15.32	<0.001
Partially immunized	26.88	22.37–31.92	14.51	11.27–18.50	−12.36	<0.001
Unimmunized (zero doses)	3.29	1.92–5.60	0.34	0.11–0.98	−2.96	<0.001
Pentavalent (DPT-HepB-Hib) 3rd dose	83.26	78.65–87.04	94.07	91.15–96.07	10.81	<0.001
Measles 1st dose (by 12 months)	76.95	71.80–81.40	92.12	88.71–94.56	15.16	<0.001
Measles 2nd dose (by 24 months)	50.77	44.79–56.73	71.03	66.17–75.44	20.26	<0.001
Hepatitis B birth dose (within 7 days)	69.32	63.54–74.54	65.53	59.36–71.21	−3.79	0.358
Polio (OPV) 3rd dose	82.20	77.36–86.20	93.79	91.33–95.58	11.58	<0.001
Pneumococcal (PCV) 3rd dose	82.60	78.18–86.29	93.42	90.51–95.49	10.82	<0.001
Inactivated Polio Vaccine (IPV) 2nd dose	44.64	37.09–52.44	76.42	71.86–80.44	31.78	<0.001
Typhoid conjugate vaccine (TCV)	62.86	56.66–68.66	83.87	79.57–87.42	21.02	<0.001
Child has an immunization card (seen)	69.94	64.15–75.16	84.27	81.19–86.94	14.33	<0.001

** Age-appropriate immunization as per Expanded Program on Immunization (EPI), Pakistan (BCG, OPV0, OPV 1–3, Penta 1–3, PCV 1–3, IPV1, Measles1), considered fully immunized; CI: Confidence interval.

**Table 3:** Full immunization coverage across subgroups.

	Baseline		Endline		Change (pp)	*p*-Value
	Coverage (%)	95% CI	Coverage (%)	95% CI		
**Residence**						
Peri-urban	67.56	51.45–80.37	81.18	67.19–90.08	13.62	.ref
Urban	70.29	64.47–75.52	86.30	82.44–89.42	16.01	0.649
**Gender**						
Male	68.51	61.55–74.74	83.10	78.03–87.20	14.59	.ref
Female	71.23	64.67–77.00	87.34	82.22–91.15	16.11	0.434
**Maternal Education**						
No formal schooling	45.70	36.24–55.48	72.09	60.23–81.51	26.40	.ref
Primary school	59.08	40.48–75.39	84.37	74.05–91.08	25.30	0.720
Middle school	61.47	49.89–71.88	91.66	83.03–96.10	30.19	0.169
Secondary (Matric)	74.18	67.35–80.00	87.20	81.41–91.38	13.03	0.519
Higher secondary or above	80.82	75.00–85.56	88.05	83.47–91.49	7.23	0.189
**Wealth Quintile**						
Poorest	48.92	38.57–59.37	76.55	65.81–84.69	27.62	.ref
Poor	68.56	58.48–77.15	83.21	74.58–89.33	14.65	0.387
Middle	73.03	64.88–79.87	87.59	81.50–91.87	14.56	0.541
Rich	73.56	63.61–81.58	90.25	84.16–94.16	16.68	0.960
Richest	81.55	72.08–88.32	87.72	81.14–92.22	6.17	0.135

CI: Confidence interval; pp: percentage point; .ref denotes the reference category against which other groups in the same variable are compared.

## Data Availability

The datasets used and/or analysed during the current study are available from the corresponding author on reasonable request.

## Supplementary Materials

The following supporting information can be downloaded at: https://www.mdpi.com/article/10.3390/vaccines14020167/s1, Table S1: Penta 3 and Measles 1 coverage across subgroups.

## Abbreviations

The following abbreviations are used in this manuscript:

BCG	Bacille Calmette–Guérin Vaccine
CI	Confidence Interval
DPT	Diphtheria–Pertussis–Tetanus
EMR	Electronic Medical Record
EPI	Expanded Programme on Immunization
FIC	Fully Immunized Children/Full Immunization Coverage
GIS	Geographic Information System
GPEI	Global Polio Eradication Initiative
HepB	Hepatitis B
Hib	Haemophilus influenzae type b
HMIS	Health Management Information System
IPV	Inactivated Polio Vaccine
LMIC	Low- and Middle-Income Countries
MR	Measles-Rubella Vaccine
NIDs	National Immunization Days
NSTOP	National Stop Transmission of Polio
OPV	Oral Polio Vaccine
PCV	Pneumococcal Conjugate Vaccine
PEI	Polio Eradication Initiative
Penta-3	Pentavalent Vaccine Third Dose (DPT-HepB-Hib)
PSU	Primary Sampling Unit
RI	Routine Immunization
TCV	Typhoid Conjugate Vaccine
TPVICS	Third-Party Verification of Immunization Coverage Survey
WHO	World Health Organization
