# Supplementary material for: A Scalable Polio-EPI Synergy Model for Urban Immunization: Coverage Gains Following Workforce Integration in Lahore, Pakistan
Source: Vaccines (Basel). 2026 Feb 11;14(2):167. doi: 10.3390/vaccines14020167 (PMC12945049; doi:10.3390/vaccines14020167)
Supplement: Supplementary file 1 [file vaccines-14-00167-s001.zip › vaccines-4116945-supplementary.pdf]

Table S1. Penta 3 and Measles 1 coverage across subgroups

Supplementary	Penta 3						Measles 1					
	Baseline		Endline		Change (pp)	P-value	Baseline		Endline		Change (pp)	P-value
	Coverage (%)	95% CI	Coverage (%)	95% CI			Coverage (%)	95% CI	Coverage (%)	95% CI		
**Residence**												
Peri-urban	81.88	71.87 - 88.88	89.07	77.08 - 95.18	7.19	.ref	73.46	59.97 - 83.64	86.34	72.61 - 93.78	12.88	.ref
Urban	83.54	78.40 - 87.65	95.51	93.54 - 96.90	11.97	0.162	77.65	72.28 - 82.24	93.79	91.38 - 95.55	16.13	0.279
**Gender**												
Male	81.42	75.10 - 86.43	93.61	90.51 - 95.74	12.19	.ref	75.59	69.24 - 80.99	91.86	87.95 - 94.58	16.27	.ref
Female	85.21	80.61 - 88.87	94.56	90.54 - 96.93	9.35	0.766	78.40	72.22 - 83.51	92.39	87.68 - 95.40	14.00	0.804
**Maternal Education**												
No formal schooling	61.71	52.48 - 70.17	84.15	73.18 - 91.17	22.43	.ref	52.91	43.37 - 62.25	81.28	70.76 - 88.62	28.36	.ref
Primary school	72.29	53.94 - 85.32	91.93	83.40 - 96.27	19.64	0.653	72.16	57.70 - 83.12	90.89	81.77 - 95.69	18.73	0.995
Middle school	83.38	70.29 - 91.41	96.05	89.13 - 98.63	12.67	0.620	75.13	61.11 - 85.31	92.97	84.68 - 96.94	17.84	0.846
Secondary (Matric)	88.82	82.56 - 93.02	97.65	94.59 - 99.00	8.83	0.451	82.84	75.09 - 88.55	94.47	90.11 - 96.98	11.63	0.846
Higher secondary or above	91.46	86.58 - 94.67	96.27	93.18 - 97.99	4.81	0.593	85.94	80.78 - 89.89	95.65	92.76 - 97.42	9.70	0.884
**Wealth Quintile**												
Poorest	66.11	55.96 - 74.96	85.88	74.14 - 92.80	19.77	.ref	56.36	46.59 - 65.66	82.76	71.02 - 90.38	26.39	.ref
Poor	86.09	78.28 - 91.40	95.82	91.47 - 98.00	9.73	0.762	78.91	70.01 - 85.71	95.36	90.92 - 97.68	16.45	0.468
Middle	87.89	80.66 - 92.67	94.43	90.19 - 96.91	6.54	0.637	80.42	72.84 - 86.29	92.43	86.59 - 95.85	12.01	0.677
Rich	83.11	74.70 - 89.13	96.87	92.93 - 98.64	13.76	0.294	77.48	68.19 - 84.66	95.20	90.06 - 97.74	17.72	0.464
Richest	91.02	81.65 - 95.85	96.87	93.48 - 98.53	5.85	0.978	88.36	78.80 - 93.94	94.27	88.25 - 97.30	5.91	0.426
